# Health services utilization and associated factors among fee waiver beneficiaries’ in Dessie city administration, Northeast Ethiopia: a cross-sectional study design

**DOI:** 10.1186/s12913-022-08963-7

**Published:** 2022-12-17

**Authors:** Nigusie Tadesse, Amsalu Feleke, Muluken Genetu Chanie, Kidist Adamu, Asnakew Molla Mekonen

**Affiliations:** 1 Department of Health Systems and Management, School of Public Health, College of Medicine and Health Sciences, Wollo University, Dessie, Ethiopia; 2 Department of Health Systems and Policy, Institute of Public Health, College of Medicine and Health Sciences, University of Gondar, Gondar, Ethiopia

**Keywords:** Fee waiver beneficiaries, Health service utilization, Dessie, Ethiopia

## Abstract

**Background:**

The fee waiver system is one of the components of the 2004 health care financing reform in Ethiopia. It is a system for granting access to health services to those who are unable to pay. The utilization health services among fee waiver beneficiaries remain low and unevenly distributed. This study aimed to assess the utilization of health services and associated factors among fee waiver beneficiaries in Dessie City, Northeast Ethiopia.

**Methods:**

A community-based cross-sectional study design was employed in Dessie City from March 23 to April 23, 2021. The study was conducted among 407 fee waiver beneficiaries. A structured, interviewer-administered questionnaire was used to collect data. Participants were selected using a simple random sampling technique. Both bi-variable and multi-variable binary logistic regressions were performed. Significant factors for the outcome variable were identified at 95% CI with a *p*-value < 0.05.

**Results:**

The overall health service utilization among fee waiver beneficiaries was found to be 62.4% (95% CI: 58.1–67.2). Being an urban resident [AOR:2.83, 95% CI:1.26–6.32], having a merchant occupation [AOR:0.20, 95% CI:0.05–0.80], having an average monthly income of 500–1000 birr [AOR:3.22, 95% CI:1.06–6.90], having a chronic disease [AOR:8.36, 95% CI:4.47–15.62], and perceiving the severity of illness as mild [AOR: 0.24, 95% CI: 0.07–0.81] were found to be statistically associated factors with health service utilization.

**Conclusions:**

The fee waiver beneficiaries were not fully utilizing health services at public health facilities. Being an urban resident, being a merchant, having an average monthly income of 500–1000 birr, having a chronic disease, and perceived severity of illness were significantly associated with health service utilization. As a result, boosting income-generating strategies and urbanizing rural parts of the city may improve health service utilization among fee waiver beneficiaries.

**Supplementary Information:**

The online version contains supplementary material available at 10.1186/s12913-022-08963-7.

## Background

Around 150 million people worldwide suffer from financial catastrophe annually, and another 100 million are pushed below the poverty line. As a result, there is a considerable risk of financial disaster and destitution, making universal health coverage (UCH) difficult to achieve [1, 2]. Besides, for a variety of reasons, more than one billion people, mostly in low and middle-income countries (LMICs), are unable to obtain essential healthcare services. Due to differences in service availability and efficiency of public health facilities, health service utilization varies greatly among countries [3]. Moreover, evidence from Ethiopia shows that health service utilization among the poor remains low and unevenly distributed [4].

Ethiopia has implemented various reforms to improve the accessibility and quality of health services. In 1998, a health care financing strategy was established and envisioned a wide range of reform initiatives [5]. In 2004, actual implementation was initiated in Amhara, Oromia, and Southern Nations, Nationalities, and People (SNNP) regional states following the ratification and endorsement of regional proclamations, regulations, and directives by the respective regional councils (parliaments), regional executive councils (cabinets), and regional health bureaus (RHBs). Currently, the reforms have expanded to the remaining regions [6]. The health-care financing reform includes revenue retention and use at the health-care facility level; systematizing a fee waiver system for the poor; standardizing exemption services for all; setting and revising user fees; establishing a private wing in public hospitals; outsourcing non-clinical services; and promoting health-care facility autonomy through the establishment of a governance body [7].

The fee waiver system is used to ensure that health-care services are available to those in society who cannot afford them [8, 9]. Since the implementation of the fee waiver, an increasing number of poor households have experienced better access to health services compared with out-of-pocket (OOP) payers [10]. The government allocated a budget, usually at the district level, for reimbursement, and the health facilities were reimbursed on the basis of a fee for service [5].

Global evidence, especially in Africa, showed that fee waiver systems did not improve health service utilization as expected [11, 12]. The fee waiver system still lacks financial security for the poor and exposes them to OOP expenses in Ethiopia [6]. According to studies conducted in different parts of Ethiopia, the highest level of health service utilization among fee waiver beneficiaries was in Gondar town (61.8%) [13], Daunt (60.98%) [14], Gamo Gofa (59.6%) [15], and Tigray (51.5%) [10].

In Ethiopia, the fee waiver system is a challenging issue that is characterized by ineffectiveness in targeting the poor, incompleteness of coverage of health services, and lack of documentation. Furthermore, due to the a lack of pharmaceuticals, innovative treatments, and laboratory services in public health facilities, the fee waiver system did not protect patients from having to pay for medicines [13, 16].

According to a study conducted in South Africa and Serbia; age, wealth status, the absence of drugs and supplies, the lack of information about waivers, the difficulty in identifying beneficiaries, and the inadequate operating structure were significant factors in health service utilization, but occupation, education, and income level were not [9, 17]. Besides studies done in Ethiopia, sex, residence, occupation status, family size, perceived health status, travel time, perceived distance, earning more than the poverty line, perceived severity of illness, shortage of drugs and procedures, and perceived transport costs were significantly associated with utilization of health services [14, 15, 18]. Additionally, studies revealed that marital status, educational status, income level, presence of a disability health problem, presence of under-five children in the household, presence of elders in the household, insurance status, the nearest health institution, the presence of chronic illness in the household, the time taken to reach the health institution, and attitude were significantly associated with the utilization of health services [16, 19–22].

Fee waiver was implemented as part of Ethiopia’s health care finance reform, but little is known about how fee waiver beneficiaries use health services, particularly in Dessie town. Thus, the study was aimed at assessing health service utilization and associated factors among fee waiver beneficiaries. The results of this study will also provide a strong hint to the relevant bodies to develop evidence-based initiatives to reduce the influence of the issue.

## Methods

### Study setting and design

A community-based, cross-sectional study was conducted in the Dessie city administration from March 23–April 23, 2021. Dessie City is 401 km away from Addis Ababa, which is the capital city of Ethiopia. Recently, the city has been organized into five sub-city administrations and eight rural kebeles. The city has an estimated total population of 201,274; of these, 104,437 were females in 2021. There are two public hospitals, three private hospitals, and eight public health centers. In addition, there are 6036 fee-waived beneficiary households [23].

### Source population

All fee waiver beneficiary households found in the Dessie City Administration were the source population.

### Study population

The study populations were randomly selected fee waiver beneficiary households. All members of the fee waiver beneficiary households whose age was > = 18 years old and who had been members for at least 12 months prior to the data collection period were included. But those who were unable to communicate and had no home-based caregivers during the data collection period were excluded.

### Sample size determination and sampling technique

The total sample size (n) was calculated using a single population proportion formula and assuming the proportion of health services utilization among fee waiver beneficiaries (59.6%) [15], a 95% confidence level, a 5% margin of error, and finally adding a 10% non-response rate, which yielded a total sample size of 407.

A stratified sampling technique was employed for this study. Eight rural kebeles and 18 urban kebeles in five sub-cities were stratified into rural and urban strata. In 18 urban kebeles, 5534 households were found and in 8 rural kebeles, 502 fee waiver beneficiaries were found. From urban (373) and rural (34) households, samples were taken through proportional allocation using a simple random sampling technique. A list of the households with their respective addresses was available in each kebele, and the list was used as a reference frame to employ a simple random sampling technique. The data were collected from one member of the household, whose age is greater than equal to 18 years old. For members of households who were absent during the data collection period, the collectors revisited the households and took the data.

### Data collection tools

Data were collected using pre-tested interviewer-administered questionnaires that were adapted from previous studies [15, 18, 19] and the World Health Organization manual for the household survey to measure access to and use of medicines [24]. The six trained data collectors (three nurses, an environmental health professional, and two midwives) were recruited for data collection. Predisposing factors, need factors, and enabling factors were included in the questionnaire. The questionnaires were prepared in English and were translated to the local language, Amharic, and translated back to English after data collection by language experts. Six data collectors had gotten a-one day training by the principal investigators about the study and how to approach respondents, the inclusion and exclusion criteria, and the operational definition of variables.

### Study variables and operational definitions

Health service utilization was the dependent variable. Independent variables were predisposing factors (sex, age, residence, marital status, religion, occupational status, educational status, family size), enabling factors (availabilities of drugs, availability of laboratory services, delayed renewal of waiver certificates, waiting time, access to referral to a higher institution, distance from the health institution, income, and non-medical costs (transport, food, and lodging)), and need factors (perceived severity of illness, perceived health status, chronic health problem, bureaucracy at the health facility and kebeles, and number of illness).

Health service utilization is operationalized as the utilization of available healthcare services in a health facility for seeking medical care in the last 12 months prior to the study. It was a dichotomized variable based on the survey question, “Did you go for health care in the last 12 months?” Yes =1, and No = 0 [18]. A fee waiver is an exemption from the requirement to pay for health care services for those who cannot afford them [21]. Beneficiaries are the poor who have been identified for use health services without charging or payment [21]. Perceived health status is the participants’ report about their health status that was assigned numerical values according to the following Likert scale: Very good = 5, good = 4, medium =3, poor =2, and very poor = 1 [22]. Perceived severity of illness is the respondents’ report about the severity of illness that was assigned numerical values according to the following Likert scale: very severe = 4, severe = 3, moderate = 2, mild = 1 [18]. The waiting time is the time from the arrival of the client at the health facility until the reception of the service [25]. Drug availability refers to the availability of all prescribed drugs in public health facilities for use by fee waiver beneficiaries [25]. Availability of laboratory services is the availability of all ordered laboratory requests in the public health facilities to be used by fee waiver beneficiaries [25]. Bureaucracy refers to uncooperativeness, negligence, and dalliance in providing health care services and long waiting times at health facilities and kebeles. An annual income of less than 7184 Birr per person per year is below the poverty line. Above poverty line is an annual income greater than 7184 Birr per person per year [26]. “Kebele” is the smallest administrative unit, which comprises around 5000 people.

### Data quality control

Before data collection, all of the data collectors were trained to have a similar idea on the questionnaire. After data collection, the collected data were checked for completeness and correctness of the data. The questionnaire was pre-tested on 50 study participants at Kombolcha city to check for understandability and corrected accordingly.

### Data analysis

Data were checked, coded and entered to EPI-data version 4.6 and exported to Statistical Package for Social Science (SPSS) version 26 for analysis. Descriptive summaries like mean, tables and figures were used to describe the study variables.

Binary logistic regression was used to identify factors associated with health service utilization among fee waiver beneficiaries’. The model goodness of fit was checked with the Hosmer-Lemeshow test (*p* = 0.51). Independent variables with a *p*-value < at 0.20 in the bi-variable analysis were considered for multivariable logistic regression analysis. Significant factors were interpreted using an adjusted odds ratio with a 95% confidence level and a *p*-value less than 0.05.

## Results

### Socio-demographic and economic characteristics of the study participants

A total of 399 households participated in this study, with a response rate of 98.03%. Most of the participants, 82% were from urban areas. Of the total participants, 64.4% were female. The median age of the participants was 48 years, with an interquartile range of 38–64 years. One hundred fifty-five (38.8%) of the participants were married, and 45.6% of the participants were Muslim. Regarding educational status, 42.1% participants can read and write, and out of the total participants, 43.9% participants are daily laborers. Three hundred forty-eight (87.2%) of the respondents had less than or equal to five family members. The median income of study participants was 1600 birr, with an interquartile range 1240–1900 birr (Table 1).

**Table 1:** Socio-demographic and economic characteristics of fee waiver beneficiaries in Dessie City Administration, Northeast Ethiopia, 2021(*n* = 399)

Characteristics	Category	Frequency ***(n)***	Percentage ***(%)***
Sex	Male	142	35.6
	Female	257	64.4
Age (in years)	18–24	22	5.5
	25–50	209	52.4
	> 50	168	42.1
Marital status	Single	31	7.8
	Married	155	38.8
	Divorced	112	28.1
	Widowed	101	25.3
Religion	Orthodox Christian	138	34.6
	Muslim	182	45.6
	Protestant	59	14.8
	Catholic	20	5.0
Educational status	Unable to read and write	46	11.5
	Read and write	168	42.1
	Elementary	115	28.8
	Secondary school and above	70	17.5
Occupational status	Farmer	35	8.8
	House wife	117	29.3
	Merchant	23	5.8
	Daily laborer	175	43.9
	Retired	49	12.3
Family size	< 5	348	87.2
	> = 5	51	12.8
Place of residence	Urban	327	82.0
	Rural	72	18.0
Monthly income (in ETB)	500–1000	26	6.5
	1001–2000	319	79.9
	> 2000	54	13.3

### Utilization of health care services by fee waiver beneficiaries

**Fig. 1:**
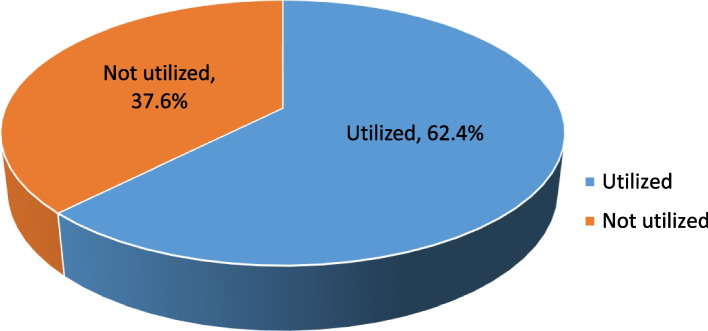
Level of health service utilization among the fee waiver beneficiaries in Dessie City Administration, Northeast, Ethiopia**,** 2021 (*n* = 399)

The overall health service utilization among fee waiver beneficiaries was found to be 62.4% (95% CI: 58.1–67.2) (Fig. 1).

### Need related factors of the study participants

All health service-utilized study participants (100%) visited government health facilities. Of these, 43.1% had chronic diseases. The average number of visits to the health facilities for the last 12 months is 2.5, with a minimum of 1 and a maximum of 4 visits. Out of all the study participants who utilized health services, 64.7% couldn’t get all the treatment they needed at a government health facility. The presence of bureaucracy at health facilities was the main reason for not getting access to referrals to higher institutions among fee waiver beneficiaries. Furthermore, self-medication (40%) was cited as a reason for not visiting a health facility during an illness (Table 2).

**Table 2:** Need related factors among fee waiver beneficiaries in Dessie City Administration, Northeast Ethiopia, 2021 (*n* = 249)

Variables	Category	Frequency	Percentage
Types of health facilities visited by fee waiver beneficiaries	Government	249	100.0
	Private	0	0
Frequency of visiting health facility to get health services	1 time	52	20.8
	2 times	106	42.6
	3 times	71	28.5
	> = 4 times	20	8.0
Getting all the treatment needed in governmental health facilities	Yes	88	35.3
	No	161	64.7
Health service utilization sites	Hospitals	15	17.0
	Health centers	73	83.0
Admitted to a health facility for any reason during the last 12 months	Yes	102	41.0
	No	147	59.0
Getting access to referral to higher health facilities	Yes	18	7.2
	No	231	92.8
Reason for not getting access to referral	I am not eligible for referral	58	25.1
	Bureaucracy at health facility	64	27.7
	Shortage of money for transportation	109	47.2
Common reasons for not visiting the health facilities	Distance from health facility	10	6.7
	Long waiting time	20	13.3
	Self-medication	60	40.0
	Unavailability of drugs	26	17.3
	Considering the disease was not sever	22	14.7
	Shortage of money for transportation	12	11.3
Presence of chronic disease	Yes	172	43.1
	No	227	56.9
Number of chronic disease	1	25	14.5
	> = 2	147	85.5

### Perceived severity of illness

**Fig. 2:**
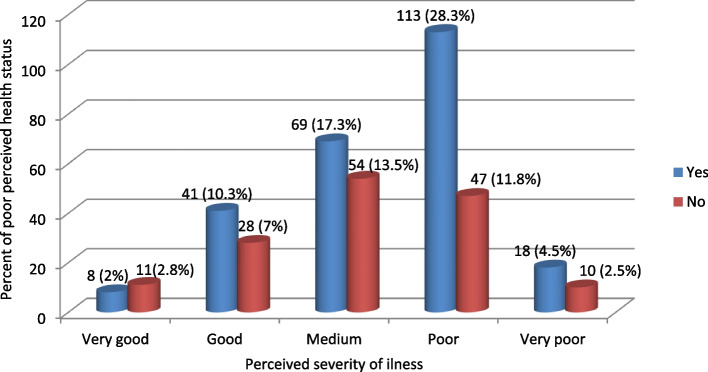
Perceived severity of illness among fee waiver beneficiaries in Dessie City Administration, Northeast, Ethiopia, 2021 (*n* = 399)

Of all the study participants, 33.1% perceived the severity of the illness as severe. On the other hand, only 4.8% of the study participants perceived the severity of the illness as mild (Fig. 2).

### Enabling factors of the study participants

Of all participants, 83% reached the nearest health facility in less than or equal to 1 h, and 71.2% used a vehicle as a means of transportation. The median waiting time of the participants was 120 minutes, with an interquartile range of 60–240 minutes. One hundred sixty-four (41.1%) of the study participants reported that the waiting time after reaching the health facility was greater than 60 minutes. Among study participants, 45.9% perceived that the time spent at health facilities was long. Only 37.3% of participants received all of the ordered laboratory services at governmental health institutions (Table 3).

**Table 3:** Enabling factors among fee waiver beneficiaries in Dessie City Administration, Northeast, Ethiopia, 2021 (*n* = 399)

Variables	Category	Frequency	Percentage
Time to reach the nearest health facility	≤ 1 hour	331	83.0
	> 1 hour	68	17.0
Way of transportation	Foot	115	28.8
	Vehicle	284	71.2
Perceived distance to nearest health facility	Very near	23	5.8
	Near	159	39.8
	Medium	131	32.8
	Far	86	21.6
Perceived transportation cost	Expensive	145	36.3
	Medium	228	57.1
	Cheap	26	6.5
Waiting time after reaching to health institution	< 30 minute	29	7.3
	30–60 minute	56	14.0
	> 60 minute	164	41.1
Perceived time spent at health facility	Long	183	45.9
	Short	42	10.5
	Appropriate	24	6.0
Availability of prescribed drugs in public health facilities	Yes all	65	16.3
	Partially	177	44.4
	Not at all	7	1.8
Condition of payment (drugs)	Paid total amount	6	1.5
	Paid partial amount	177	44.4
	Did not pay	64	16.0
Availability of ordered laboratory services in public health facilities	Yes all	95	23.8
	Partially	149	37.3
	Not at all	4	1.3
Condition of payment (laboratory services)	Paid total amount	7	1.8
	Paid partial amount	148	37.1
	Did not pay	94	23.6
Certificate renewal in this year	Yes	389	97.5
	No	10	2.5
Difficulty in renewing waiver certificate	Yes	49	12.3
	No	350	87.7

### Factors associated with health service utilization among fee waiver beneficiaries

Both bi-variable and multivariable logistic regressions were done to identify factors associated with health service utilization among fee waiver beneficiaries. During bi-variable analysis, twelve variables (sex, religion, occupational status, place of residence, family size, monthly income, perceived health status, having chronic disease, perceived severity of illness, perceived distance to a nearby health facility, perceived transportation cost, and distance from a nearby health facility) were statistically significant with regard to health service utilization and were included in multivariable logistic regression.

In multivariable logistic regression, place of residence, occupational status, average monthly income, having a chronic disease, and perceived severity of illness were found to be statistically significant with regard to health service utilization among fee-waiver beneficiaries.

Urban residents were 2.83 times more likely to utilize health services as compared to rural residents [AOR: 2.83, 95% CI: 1.26–6.32]. Participants who were merchants were 0.20 times less likely to utilize health services as compared to those who were retired [AOR: 0.20, 95% CI: 0.05–0.80]. Participants who had an average monthly income of between 500 and 1000 birr were 3.22 times more likely to utilize health care services as compared to those who had an average monthly income of greater than 2000 birr [AOR: 3.22, 95% CI: 1.06–6.90]. Participants having a chronic disease were 8.36 times more likely to utilize health services than those without a chronic disease [AOR: 8.36, 95% CI: 4.47–15.62]. Participants who perceived the severity of illness as mild were 0.24 times less likely to utilize health care services as compared to those who perceived the severity of illness as very severe [AOR: 0.24, 95% CI: 0.07–0.81] (Table 4).

**Table 4:** Factors associated with health service utilization among fee waiver beneficiaries in Dessie City Administration, Northeast, Ethiopia, 2021 (*n* = 399)

Variables	Category	Health Service Utilization		COR (95%CI)	AOR (95%CI)
		Yes	No		
Place of residence	Urban	102	225	1.23(1.03–3.39)	2.83(1.26–6.32)*
	Rural	48	24	1	1
Occupational status	Farmer	13	22	0.17(0.66–0.45)	1.25(0.33–4.77)
	House wife	66	51	0.375(0.17–0.80)	0.54(0.19–1.54)
	Merchant	10	13	0.22(0.08–0.65)	0.20(0.05–0.80)*
	Daily laborer	122	53	0.66 (0.32–1.40)	0.48 (0.18–1.27)
	Retired	38	11	1	1
Monthly income (ETB)	500–1000	13	13	2.15 (1,05–4.45)	3.22 (1.06–6.90)*
	1001–2000	189	130	0.217 (0.10–0.49)	0.67 (0.23–1.94)
	> 2000	47	7	1	1
Having chronic disease	Yes	151	21	9.47 (5.59–16.03)	8.36 (4.48–15.62)***
	No	98	129	1	1
Perceived severity of illness	Mild	19	23	0.41(0.16–1.09)	0.24 (0.07–0.81)*
	Moderate	78	61	0.64 (0.28–1.47)	0.40 (0.14–1.16)
	Sever	132	56	1.18 (0.52–2.68)	0.52 (0.18–1.49)
	Very sever	20	10	1	1

Note: *COR* Crude 0dds Ratio, *AOR* Adjusted Odds Ratio, *CI* Confidence Interval, *(*p* < 0.05), *** (*p* < 0.0001)

## Discussion

In this study, the overall health service utilization was 62.4%. Additionally, place of residence, occupational status, monthly income, having a chronic disease, and perceived severity of illness were significantly associated with health service utilization.

In this study, the level of health service utilization was higher when compared with studies conducted in Southwest Ethiopia (45.6%), North Ethiopia (51.5%), Northeast Ethiopia (41.8%), Southern Ethiopia (59.6%), and the Northwest (29.3%) [10, 15, 18, 19, 22]. This could be a difference in the implementation of the fee waiver system across Ethiopia; the fee waiver is fully implemented in Dessie City compared with other areas. Our study showed that all of the study participants (100%) visited public health facilities. But it is in line with the studies conducted in Northwest Ethiopia (61.8%) and Northeast Ethiopia (60.98%) [13, 14]. This could be due to similarities in the implementation of the fee waiver system; both study areas are found in the same region.

Being an urban resident was also positively related to health service consumption, which is consistent with a study conducted in Northeast Ethiopia and Serbia [14, 17]. This could be because urban residents have easier access to medical information and health services.

Besides, in this study, occupation was found to be negatively related to health service utilization. It is consistent with the study in South Gondar zone [20]. This could be because merchants are too busy with their regular business activities to visit health institutions. Alternatively, their income may rise, and they may visit more private health facilities in favor of public health facilities.

The study also found that mild perceived severity of illness was associated with lower health-care utilization. It is supported by the finding in Northeast Ethiopia [18]. This is due to the fact that people do not go to health institutions unless they are seriously ill. Moreover, people do not self-refer to health services unless they are in grave danger.

Furthermore, having a chronic illness is linked to increased use of health-care services. Participants with having a chronic health problem were more likely to seek medical care compared with those without a chronic health problem. This is supported by evidence from South Africa, Northwest Ethiopia, West Ethiopia, and Northeast Ethiopia [18, 19, 22, 27]. This is the fact that patients with chronic health problems need repeated, close follow-up, which increases their medical treatment.

Participants with lower monthly incomes are more likely to use health services compared with those with higher incomes. This was supported by studies conducted in West Ethiopia [19] and Northeast Ethiopia [18]. This may be due to financial restrictions; they depend on public health facilities, which increase the utilization of health services.

### Limitations of the study

The study was done with some limitations. Results for some variables have large confidence intervals (CI), which may be a result of the small sample size, which reduces the accuracy of the findings. Due to the interviewer’s administration of the questions, some variables may be subject to social desirability bias. Recall bias may be an issue because the examination of self-reported behavior patterns was retroactive. Participants might forget parts of their experiences because we employed 1-year of recall. Additionally, income measurement, which does not account for valuable things amassed over the course of a person’s life, may not accurately reflect household wealth.

## Conclusions

The overall health service utilization was low but consistent with previous studies and affected by place of residence, occupational status, monthly income, having chronic diseases, and the perceived severity of illness. We recommended that Dessie city administration, Dessie city administration health department, and health facilities focus on boosting income-generating strategies, urbanizing rural parts of the city; and creating awareness about the severity of disease to improve health service utilization among fee waiver beneficiaries.

## Supplementary Information

**Additional file 1: Table S1.** STROBE 2007 (v4) Statement—Checklist of items that should be included in reports of cross-sectional studies.

## Data Availability

The datasets used and/or analyzed during the current study are available from the first author on reasonable request.

## Abbreviations

ANRS: Amhara National Regional State
ETB: Ethiopian Birr
EPHI: Ethiopian Public Health Institute
FMOH: Federal Ministry of Health
HCF: Health Care financing
ILO: International Labor Organization
LMICs: Low and Middle Income Countries
OOP: Out of Pocket
UCH: Universal Health Coverage
USAID: United Sates Agency for International Development.
WHO: World Health Organization
