# Supplementary material for: Health services utilization and associated factors among fee waiver beneficiaries’ in Dessie city administration, Northeast Ethiopia: a cross-sectional study design
Source: BMC Health Serv Res. 2022 Dec 17;22:1544. doi: 10.1186/s12913-022-08963-7 (PMC9759911; doi:10.1186/s12913-022-08963-7)
Supplement: Supplementary file 1 — Additional file 1: Table S1. STROBE 2007 (v4) Statement—Checklist of items that should be included in reports of cross-sectional studies. [file 12913_2022_8963_MOESM1_ESM.docx]

**S1 table: STROBE 2007 (v4) Statement—Checklist of items that should be included in reports of cross-sectional studies**

**Title of the study:** Health services utilization and associated factors among fee waiver beneficiaries’ in Dessie city administration Northeast Ethiopia: A cross-sectional study design

| **Section/Topic** | | Item No. | Recommendation | Report checking |
| --- | --- | --- | --- | --- |
| **Title and abstract** | | 1 | (*a*) Indicate the study’s design with a commonly used term in the title or the abstract | The type of the study design is indicated in the tile. |
|  |  |  | (*b*) Provide in the abstract an informative and balanced summary of what was done and what was found | The abstract gives informative summary of the study. |
| Introduction | | | |  |
| Background/rationale | | 2 | Explain the scientific background and rationale for the investigation being reported | Background and rational of the study described. |
| Objectives | | 3 | State specific objectives, including any pre-specified hypotheses | The specific objectives of the study reported at the background section. |
| Methods | | | |  |
| Study design | 4 | | Present key elements of study design early in the paper | Key elements of the study design reported |
| Setting | 5 | | Describe the setting, locations, and relevant dates, including periods of recruitment, exposure, follow-up, and data collection | Setting, location, and dates reported |
| Participants | 6 | | (*a*) Give the eligibility criteria, and the sources and methods of selection of participants | Eligibility criteria, and the sources and methods of selection of participants described |
| Variables | 7 | | Clearly define all outcomes, exposures, predictors, potential confounders, and effect modifiers. Give diagnostic criteria, if applicable | Outcome (dependent variable), predictors (independent variables) reported. |
| Data sources/ measurement | 8* | | For each variable of interest, give sources of data and details of methods of assessment (measurement). Describe comparability of assessment methods if there is more than one group | Pre-disposing factors, enabling factors and need factors are described. |
| Bias | 9 | | Describe any efforts to address potential sources of bias | Reporting bias deriving from incomplete data was addressed by rejecting these data. |
| Study size | 10 | | Explain how the study size was arrived at | The total sample size (n) was calculated using a single population proportion formula and assuming the proportion of health services utilization among fee waiver beneficiaries (59.6%) (14), a 95% confidence level, and a 5% margin of error, and finally adding a 10% non-response rate, which yielded a total sample size of 407. |
| Quantitative variables | 11 | | Explain how quantitative variables were handled in the analyses. If applicable, describe which groupings were chosen and why | Methods of quantitative variables handling in the analyses was described. |
| Statistical methods | 12 | | (*a*) Describe all statistical methods, including those used to control for confounding | All statistical methods, including those used to control for confounding were reported. |
|  |  | | (*b*) Describe any methods used to examine subgroups and interactions | Not applicable |
|  |  |  | (*c*) Explain how missing data were addressed | Not applicable |
|  |  |  | (*d*) If applicable, describe analytical methods taking account of sampling strategy | Not applicable |
|  |  |  | (*e*) Describe any sensitivity analyses | Not applicable |
| **Results** |  | |  |  |
| Participants | 13* | | (a) Report numbers of individuals at each stage of study—e.g. numbers potentially eligible, examined for eligibility, confirmed eligible, included in the study, completing follow-up, and analyzed | Number of participants is stated. |
|  |  | | (b) Give reasons for non-participation at each stage | Not applicable |
|  |  | | (c) Consider use of a flow diagram | Not applicable |
| Descriptive data | 14* | | (a) Give characteristics of study participants (e.g. demographic, clinical, social) and information on exposures and potential confounders | Socio-demographic characteristics of the study participants are reported. |
|  |  | | (b) Indicate number of participants with missing data for each variable of interest | Not applicable |
| Outcome data | 15* | | Report numbers of outcome events or summary measures | Health service utilization among fee waiver beneficiaries was found to be 62.4 %. |
| Main results | 16 | | (*a*) Give unadjusted estimates and, if applicable, confounder-adjusted estimates and their precision (e.g. 95% confidence interval). Make clear which confounders were adjusted for and why they were included | Unadjusted (COR) and adjusted (AOR) estimates and their precision are described. |
|  |  | | (*b*) Report category boundaries when continuous variables were categorized | Not applicable |
|  |  | | (*c*) If relevant, consider translating estimates of relative risk into absolute risk for a meaningful time period | Not applicable |
| Other analyses | 17 | | Report other analyses done—e.g. analyses of subgroups and interactions, and sensitivity analyses | Not applicable |
| Discussion |  | |  |  |
| Key results | 18 | | Summarise key results with reference to study objectives | Key results with reference to study objectives are summarized. |
| Limitations | 19 | | Discuss limitations of the study, taking into account sources of potential bias or imprecision. Discuss both direction and magnitude of any potential bias | Limitations of the study were discussed. |
| Interpretation | 20 | | Give a cautious overall interpretation of results considering objectives, limitations, multiplicity of analyses, results from similar studies, and other relevant evidence | The interpretation of the results was very  cautious, given the cross-sectional nature of  the study which does not allow to infer  causality |
| Generalizability | 21 | | Discuss the generalizability (external validity) of the study results | The generalizability of the study results was discussed. |
| Other information |  | |  |  |
| Funding | 22 | | Give the source of funding and the role of the funders for the present study and, if applicable, for the original study on which the present article is based | Not applicable |

N/A, not applicable *Give information separately for cases and controls in case-control studies and, if applicable, for exposed and unexposed groups in cohort and cross-sectional studies.

Note: An Explanation and Elaboration article discusses each checklist item and gives methodological background and published examples of transparent reporting. The STROBE checklist is best used in conjunction with this article (freely available on the Web sites of PLoS Medicine at http://www.plosmedicine.org/, Annals of Internal Medicine at http://www.annals.org/, and Epidemiology at http://www.epidem.com/). Information on the STROBE Initiative is available at www.strobe-statement.org.
